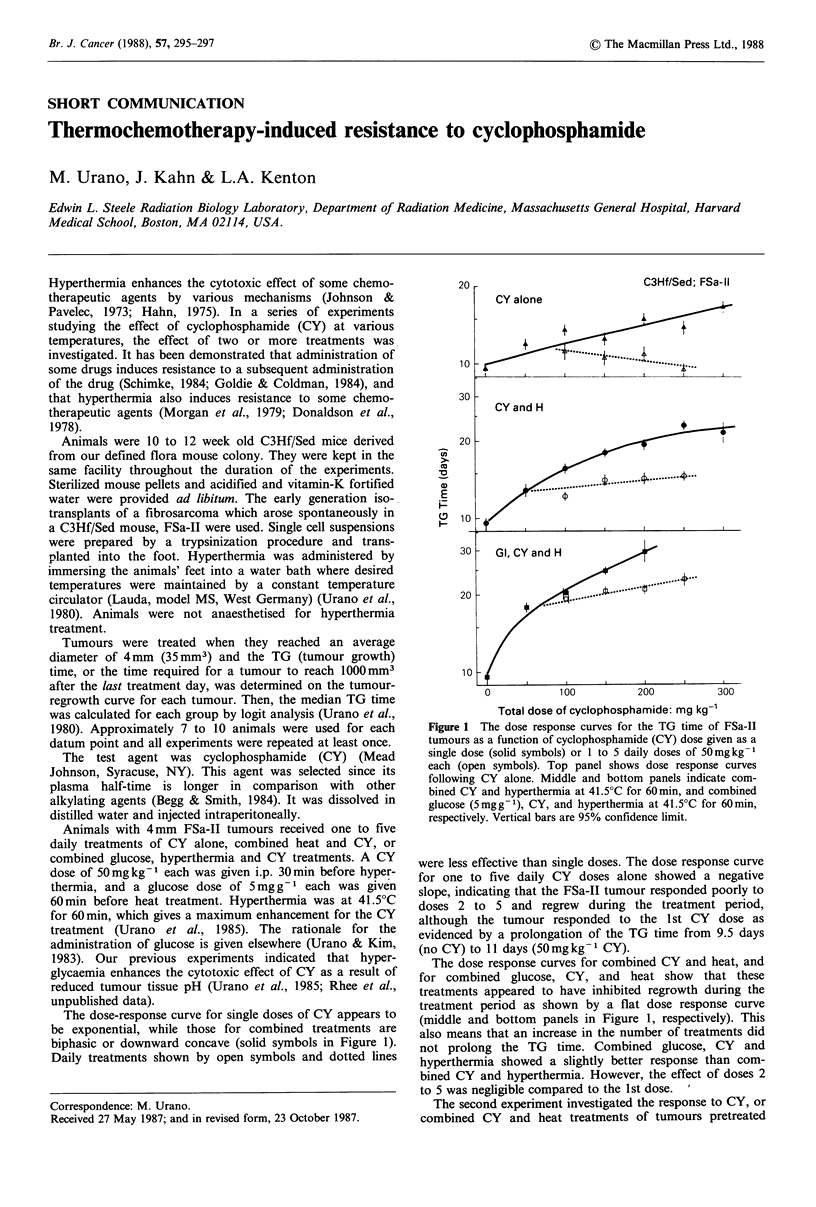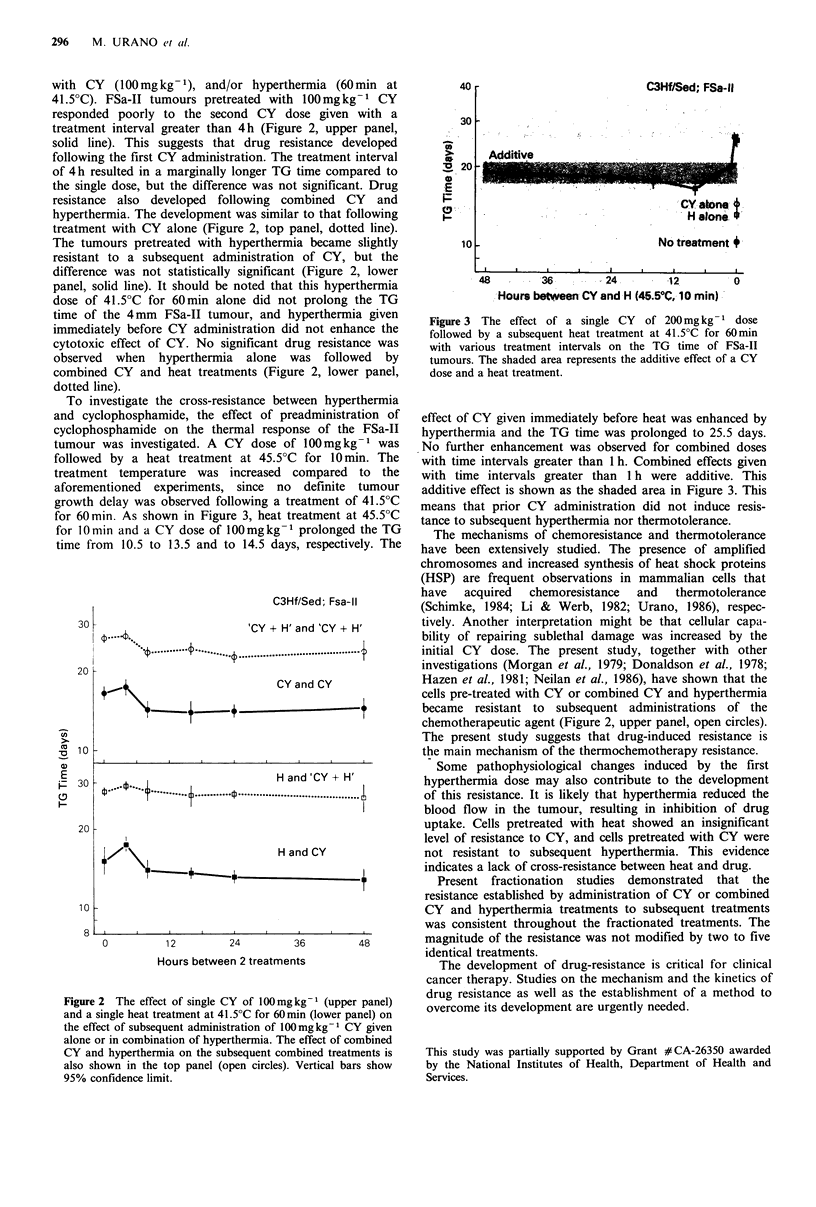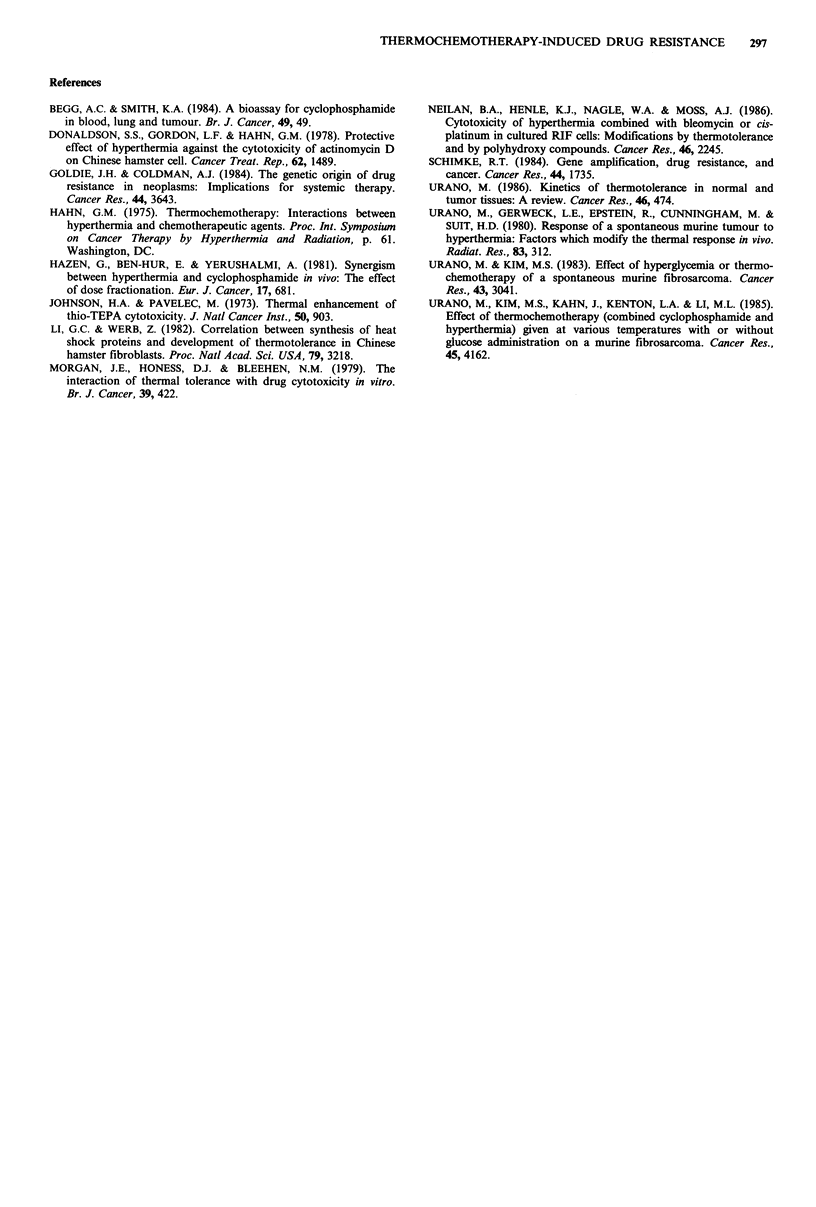# Thermochemotherapy-induced resistance to cyclophosphamide.

**DOI:** 10.1038/bjc.1988.65

**Published:** 1988-03

**Authors:** M. Urano, J. Kahn, L. A. Kenton

**Affiliations:** Edwin L. Steele Radiation Biology Laboratory, Massachusetts General Hospital, Harvard Medical School, Boston 02114.


*Br. J. Cancer* (1988), **57**, 295–297 © The Macmillan Press Ltd., 1988

SHORT COMMUNICATION

### Thermochemotherapy-induced resistance to cyclophosphamide

M. Urano, J. Kahn & L.A. Kenton

*Edwin L. Steele Radiation Biology Laboratory, Department of Radiation Medicine, Massachusetts General Hospital, Harvard Medical School, Boston, MA 02114, USA.*

Hyperthermia enhances the cytotoxic effect of some chemotherapeutic agents by various mechanisms (Johnson & Pavelec, 1973; Hahn, 1975). In a series of experiments studying the effect of cyclophosphamide (CY) at various temperatures, the effect of two or more treatments was investigated. It has been demonstrated that administration of some drugs induces resistance to a subsequent administration of the drug (Schimke, 1984; Goldie & Coldman, 1984), and that hyperthermia also induces resistance to some chemotherapeutic agents (Morgan *et al.*, 1979; Donaldson *et al.*, 1978).

Animals were 10 to 12 week old C3Hf/Sed mice derived from our defined flora mouse colony. They were kept in the same facility throughout the duration of the experiments. Sterilized mouse pellets and acidified and vitamin-K fortified water were provided *ad libitum*. The early generation isotransplants of a fibrosarcoma which arose spontaneously in a C3Hf/Sed mouse, FSa-II were used. Single cell suspensions were prepared by a trypsinization procedure and transplanted into the foot. Hyperthermia was administered by immersing the animals' feet into a water bath where desired temperatures were maintained by a constant temperature circulator (Lauda, model MS, West Germany) (Urano *et al.*, 1980). Animals were not anaesthetised for hyperthermia treatment.

Tumours were treated when they reached an average diameter of 4 mm (35 mm$^3$) and the TG (tumour growth) time, or the time required for a tumour to reach 1000 mm$^3$ after the *last* treatment day, was determined on the tumour-regrowth curve for each tumour. Then, the median TG time was calculated for each group by logit analysis (Urano *et al.*, 1980). Approximately 7 to 10 animals were used for each datum point and all experiments were repeated at least once.

The test agent was cyclophosphamide (CY) (Mead Johnson, Syracuse, NY). This agent was selected since its plasma half-time is longer in comparison with other alkylating agents (Begg & Smith, 1984). It was dissolved in distilled water and injected intraperitoneally.

Animals with 4 mm FSa-II tumours received one to five daily treatments of CY alone, combined heat and CY, or combined glucose, hyperthermia and CY treatments. A CY dose of 50 mg kg$^{-1}$ each was given i.p. 30 min before hyperthermia, and a glucose dose of 5 mg g$^{-1}$ each was given 60 min before heat treatment. Hyperthermia was at 41.5°C for 60 min, which gives a maximum enhancement for the CY treatment (Urano *et al.*, 1985). The rationale for the administration of glucose is given elsewhere (Urano & Kim, 1983). Our previous experiments indicated that hyperglycaemia enhances the cytotoxic effect of CY as a result of reduced tumour tissue pH (Urano *et al.*, 1985; Rhee *et al.*, unpublished data).

The dose-response curve for single doses of CY appears to be exponential, while those for combined treatments are biphasic or downward concave (solid symbols in Figure 1). Daily treatments shown by open symbols and dotted lines were less effective than single doses. The dose response curve for one to five daily CY doses alone showed a negative slope, indicating that the FSa-II tumour responded poorly to doses 2 to 5 and regrew during the treatment period, although the tumour responded to the 1st CY dose as evidenced by a prolongation of the TG time from 9.5 days (no CY) to 11 days (50 mg kg$^{-1}$ CY).

**Figure 1** The dose response curves for the TG time of FSa-II tumours as a function of cyclophosphamide (CY) dose given as a single dose (solid symbols) or 1 to 5 daily doses of 50 mg kg$^{-1}$ each (open symbols). Top panel shows dose response curves following CY alone. Middle and bottom panels indicate combined CY and hyperthermia at 41.5°C for 60 min, and combined glucose (5 mg g$^{-1}$), CY, and hyperthermia at 41.5°C for 60 min, respectively. Vertical bars are 95% confidence limit.

The dose response curves for combined CY and heat, and for combined glucose, CY, and heat show that these treatments appeared to have inhibited regrowth during the treatment period as shown by a flat dose response curve (middle and bottom panels in Figure 1, respectively). This also means that an increase in the number of treatments did not prolong the TG time. Combined glucose, CY and hyperthermia showed a slightly better response than combined CY and hyperthermia. However, the effect of doses 2 to 5 was negligible compared to the 1st dose.

The second experiment investigated the response to CY, or combined CY and heat treatments of tumours pretreated

Correspondence: M. Urano.
Received 27 May 1987; and in revised form, 23 October 1987.

with CY ($100 mg kg^{-1}$), and/or hyperthermia (60 min at 41.5°C). FSa-II tumours pretreated with $100 mg kg^{-1}$ CY responded poorly to the second CY dose given with a treatment interval greater than 4 h (Figure 2, upper panel, solid line). This suggests that drug resistance developed following the first CY administration. The treatment interval of 4 h resulted in a marginally longer TG time compared to the single dose, but the difference was not significant. Drug resistance also developed following combined CY and hyperthermia. The development was similar to that following treatment with CY alone (Figure 2, top panel, dotted line). The tumours pretreated with hyperthermia became slightly resistant to a subsequent administration of CY, but the difference was not statistically significant (Figure 2, lower panel, solid line). It should be noted that this hyperthermia dose of 41.5°C for 60 min alone did not prolong the TG time of the 4 mm FSa-II tumour, and hyperthermia given immediately before CY administration did not enhance the cytotoxic effect of CY. No significant drug resistance was observed when hyperthermia alone was followed by combined CY and heat treatments (Figure 2, lower panel, dotted line).

To investigate the cross-resistance between hyperthermia and cyclophosphamide, the effect of preadministration of cyclophosphamide on the thermal response of the FSa-II tumour was investigated. A CY dose of $100 mg kg^{-1}$ was followed by a heat treatment at 45.5°C for 10 min. The treatment temperature was increased compared to the aforementioned experiments, since no definite tumour growth delay was observed following a treatment of 41.5°C for 60 min. As shown in Figure 3, heat treatment at 45.5°C for 10 min and a CY dose of $100 mg kg^{-1}$ prolonged the TG time from 10.5 to 13.5 and to 14.5 days, respectively. The effect of CY given immediately before heat was enhanced by hyperthermia and the TG time was prolonged to 25.5 days. No further enhancement was observed for combined doses with time intervals greater than 1 h. Combined effects given with time intervals greater than 1 h were additive. This additive effect is shown as the shaded area in Figure 3. This means that prior CY administration did not induce resistance to subsequent hyperthermia nor thermotolerance.

**Figure 2** The effect of single CY of $100 mg kg^{-1}$ (upper panel) and a single heat treatment at 41.5°C for 60 min (lower panel) on the effect of subsequent administration of $100 mg kg^{-1}$ CY given alone or in combination of hyperthermia. The effect of combined CY and hyperthermia on the subsequent combined treatments is also shown in the top panel (open circles). Vertical bars show 95% confidence limit.

**Figure 3** The effect of a single CY of $200 mg kg^{-1}$ dose followed by a subsequent heat treatment at 41.5°C for 60 min with various treatment intervals on the TG time of FSa-II tumours. The shaded area represents the additive effect of a CY dose and a heat treatment.

The mechanisms of chemoresistance and thermotolerance have been extensively studied. The presence of amplified chromosomes and increased synthesis of heat shock proteins (HSP) are frequent observations in mammalian cells that have acquired chemoresistance and thermotolerance (Schimke, 1984; Li & Werb, 1982; Urano, 1986), respectively. Another interpretation might be that cellular capability of repairing sublethal damage was increased by the initial CY dose. The present study, together with other investigations (Morgan *et al.*, 1979; Donaldson *et al.*, 1978; Hazen *et al.*, 1981; Neilan *et al.*, 1986), have shown that the cells pre-treated with CY or combined CY and hyperthermia became resistant to subsequent administrations of the chemotherapeutic agent (Figure 2, upper panel, open circles). The present study suggests that drug-induced resistance is the main mechanism of the thermochemotherapy resistance.

Some pathophysiological changes induced by the first hyperthermia dose may also contribute to the development of this resistance. It is likely that hyperthermia reduced the blood flow in the tumour, resulting in inhibition of drug uptake. Cells pretreated with heat showed an insignificant level of resistance to CY, and cells pretreated with CY were not resistant to subsequent hyperthermia. This evidence indicates a lack of cross-resistance between heat and drug.

Present fractionation studies demonstrated that the resistance established by administration of CY or combined CY and hyperthermia treatments to subsequent treatments was consistent throughout the fractionated treatments. The magnitude of the resistance was not modified by two to five identical treatments.

The development of drug-resistance is critical for clinical cancer therapy. Studies on the mechanism and the kinetics of drug resistance as well as the establishment of a method to overcome its development are urgently needed.

This study was partially supported by Grant #CA-26350 awarded by the National Institutes of Health, Department of Health and Services.

### References

BEGG, A.C. & SMITH, K.A. (1984). A bioassay for cyclophosphamide in blood, lung and tumour. *Br. J. Cancer*, **49**, 49.

DONALDSON, S.S., GORDON, L.F. & HAHN, G.M. (1978). Protective effect of hyperthermia against the cytotoxicity of actinomycin D on Chinese hamster cell. *Cancer Treat. Rep.*, **62**, 1489.

GOLDIE, J.H. & COLDMAN, A.J. (1984). The genetic origin of drug resistance in neoplasms: Implications for systemic therapy. *Cancer Res.*, **44**, 3643.

HAHN, G.M. (1975). Thermochemotherapy: Interactions between hyperthermia and chemotherapeutic agents. *Proc. Int. Symposium on Cancer Therapy by Hyperthermia and Radiation*, p. 61. Washington, DC.

HAZEN, G., BEN-HUR, E. & YERUSHALMI, A. (1981). Synergism between hyperthermia and cyclophosphamide *in vivo*: The effect of dose fractionation. *Eur. J. Cancer*, **17**, 681.

JOHNSON, H.A. & PAVELEC, M. (1973). Thermal enhancement of thio-TEPA cytotoxicity. *J. Natl Cancer Inst.*, **50**, 903.

LI, G.C. & WERB, Z. (1982). Correlation between synthesis of heat shock proteins and development of thermotolerance in Chinese hamster fibroblasts. *Proc. Natl Acad. Sci. USA*, **79**, 3218.

MORGAN, J.E., HONESS, D.J. & BLEEHEN, N.M. (1979). The interaction of thermal tolerance with drug cytotoxicity *in vitro*. *Br. J. Cancer*, **39**, 422.

NEILAN, B.A., HENLE, K.J., NAGLE, W.A. & MOSS, A.J. (1986). Cytotoxicity of hyperthermia combined with bleomycin or *cis*-platinum in cultured RIF cells: Modifications by thermotolerance and by polyhydroxy compounds. *Cancer Res.*, **46**, 2245.

SCHIMKE, R.T. (1984). Gene amplification, drug resistance, and cancer. *Cancer Res.*, **44**, 1735.

URANO, M. (1986). Kinetics of thermotolerance in normal and tumor tissues: A review. *Cancer Res.*, **46**, 474.

URANO, M., GERWECK, L.E., EPSTEIN, R., CUNNINGHAM, M. & SUIT, H.D. (1980). Response of a spontaneous murine tumour to hyperthermia: Factors which modify the thermal response *in vivo*. *Radiat. Res.*, **83**, 312.

URANO, M. & KIM, M.S. (1983). Effect of hyperglycemia or thermochemotherapy of a spontaneous murine fibrosarcoma. *Cancer Res.*, **43**, 3041.

URANO, M., KIM, M.S., KAHN, J., KENTON, L.A. & LI, M.L. (1985). Effect of thermochemotherapy (combined cyclophosphamide and hyperthermia) given at various temperatures with or without glucose administration on a murine fibrosarcoma. *Cancer Res.*, **45**, 4162.